# Benign Mucosal Membrane Pemphigoid as a Differential Diagnosis of Necrotizing Periodontal Disease

**DOI:** 10.1155/2020/8885158

**Published:** 2020-12-22

**Authors:** Carmen Lucia Mueller Storrer, Darlan Rigo Junior, Lucimari Teixeira, Eugênio Esteves Costa, Brayan Ruppenthal Endres, Diogo Vinícius Ferreira dos Santos, Aline Monise Sebastiani

**Affiliations:** ^1^Graduate Program in Clinical Dentistry, School of Dental Health, Universidade Positivo, Curitiba, Paraná, Brazil; ^2^Private Office, Curitiba, Paraná, Brazil; ^3^School of Dental Health, Universidade Positivo, Curitiba, Paraná, Brazil

## Abstract

Desquamative gingivitis is an oral sign of systemic changes that may be symptomatic or asymptomatic. It is generally related to immunological changes. This report is aimed at describing the case of a 51-year-old female patient, who presented with clinical desquamative gingivitis and was initially diagnosed and treated as necrotizing periodontal disease, but no improvement. The cause was hypothesized to be an autoimmune disease. Drug therapy was initiated as a combination of prednisone and topical clobetasol propionate 0.5% cream twice a day. After this treatment, there was an improvement in pain and the blistering of the gingiva. However, the gingiva remained erythematous. A biopsy led to the diagnosis of benign mucous membrane pemphigoid (BMMP). The BMMP case reported in this article has been successfully treated with systemic and topical immunosuppressive therapy. The efficacy of systemic corticosteroids with dapsone and multidisciplinary follow-up has been highlighted and can lead to the stabilization and adequate control of the disease.

## 1. Background

Autoimmune diseases are characterized by an exaggerated immune response, leading to damage and dysfunction of specific or multiple organs and tissue [1]. Autoimmune blistering diseases are a rare subgroup of diseases characterized by the presence of serum autoantibodies (IgG, IgA) directed against antigens in the epithelium or basement membrane zone [2]. The oral mucosa generally represents the first site of blistering autoimmune diseases [3]. It may be the only sign in some cases, or it can spread to the skin or other places of the mucosa, such as the connective tissue, nose, pharynx, larynx, esophagus, and genital area [4].

Among autoimmune blistering diseases, benign mucous membrane pemphigoid (BMMP) is a rare autoimmune inflammatory mucocutaneous disease [3] with a higher prevalence in adult middle-aged female patients [5]. It affects the oral mucosa in most of the cases, with blisters and vesicles that rupture, producing ulcerated areas in the alveolar ridge region, jugal mucosa, and hard palate [2]. Patients may report pain on chewing, oral bleeding [2], and a history of blister formation, which may be intact on clinical examination [6]. Commonly, patients present with desquamative gingivitis, which may be confused with other periodontal changes, such as necrotizing periodontal disease (NPD) [5]. NPDs have three typical features: pain, bleeding, and ulceration of the gingival interdental papilla [7]. In these cases, Nikolsky's sign test can be used to differentially diagnose benign mucous membrane pemphigoid. This test consists of pressuring apparently normal skin or mucosa in the area adjacent to the lesion. A detachment of the superficial layer will indicate disease activity [8].

The lack of commonly recognized diagnostic criteria could result in misdiagnosis, leading to negligible treatment for this chronic and inflammatory mucous membrane group of diseases. Therefore, this report is aimed at describing the case of a 51-year-old female patient, who presented with clinical desquamative gingivitis and was initially diagnosed and treated as NPD.

## 2. Case report

A 51-year-old 63 kg female patient presented for dental care complaining of gingival bleeding, pain when eating, and oral hygiene difficulties. During the anamnesis, similar episodes were reported over the last two years, occurring after development of psychological stress. The patient presented no systemic changes and reported being postmenopausal, without climacteric symptoms, and undergoing hormonal follow-up. However, she was emotionally stressed.

**Figure 1:**
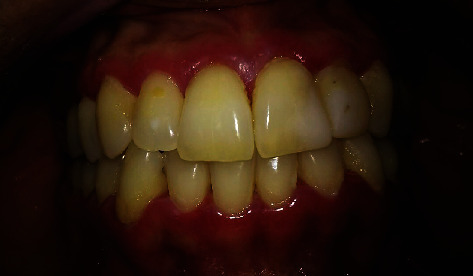
Initial characteristic of the gingiva with an erythematous aspect and with sulcus suppuration. Intraoral image of the patient at the initial examination.

Intraoral clinical examination showed exacerbated accumulation of dental biofilm, erythematous and swollen gingiva, and mild pain (Figure 1). Due to the patient's clinical characteristics, the first diagnostic hypothesis (June 15^th^) was NPD, and antibiotic therapy was proposed (amoxicillin 500 mg and metronidazole 400 mg, three times a day for seven days; chlorhexidine digluconate 0.12% mouthwash twice a day for 14 days), along with dental biofilm. At that time, the patient signed the treatment consent form.

After 14 days, no satisfactory clinical improvements were observed after the initial treatment. Even with and decreased inflammation, the gingival epithelium still had a generalized erythematous ulcerated desquamative appearance.

**Figure 2:**
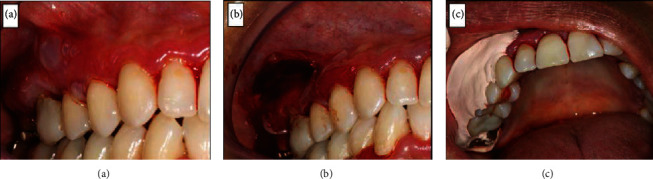
(a) Displacement of the epithelium after an air jet over the buccal gingiva, causing blisters (Nikolsky positive sign). (b) Removal of the gingival fragment. (c) Surgical cement to protect the surgical area. Intraoral images before treatment: (a) Nikolsky's sign; (b) appearance after the incisional biopsy;(c) appearance with the surgical cement.

**Figure 3:**
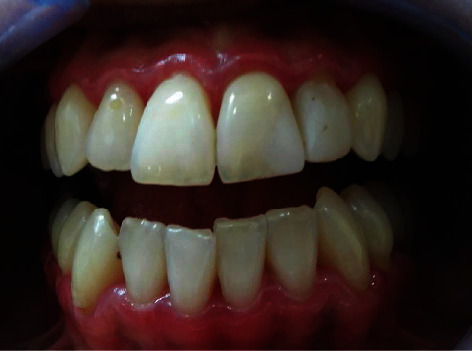
Follow-up after prednisone treatment. Intraoral image 40 days after the beginning of treatment, showing erythematous gingiva.

**Figure 4:**
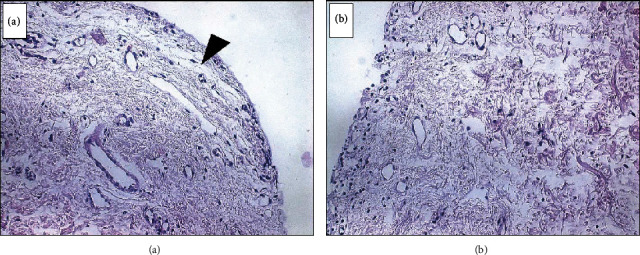
Histopathological sections of the removed tissue stained with hematoxylin and eosin. a) Slide showing the absence of epithelium with exposed connective tissue. b) Submucosal tissue showing the blistering lesion.

**Figure 5:**
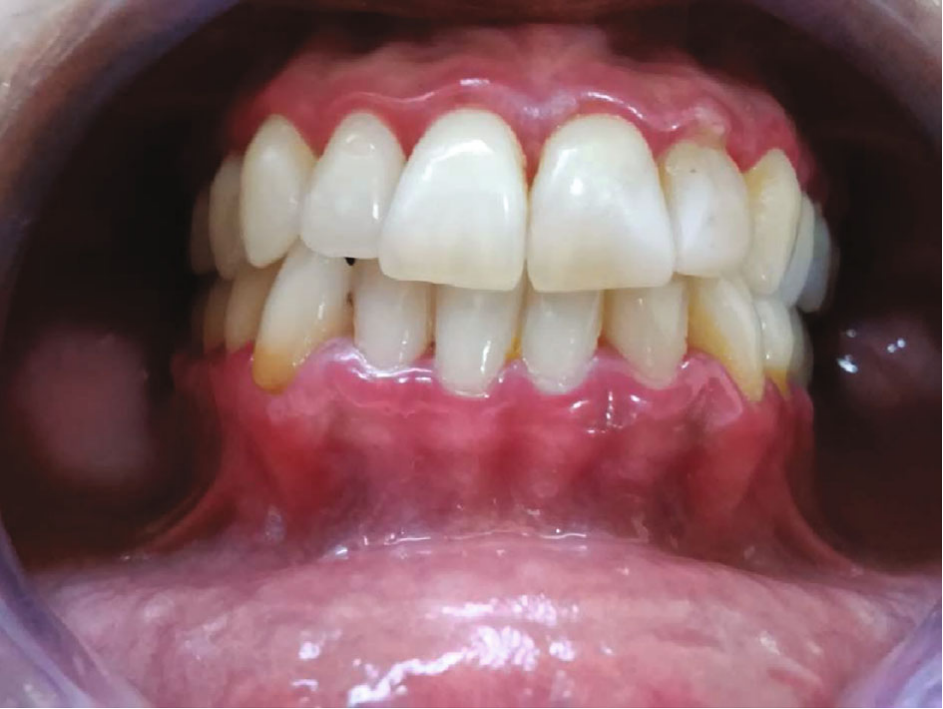
Improvement without signs of bleeding after 3 months. Photograph of intraoral aspect in the last follow-up consultation.

Indeed, the patient returned with worsened signs and symptoms and reported the emergence of “blood blisters” that burst and caused pain. The hypothesis of an immunological cause was raised as there was no clinical improvement with conventional NPD treatment. An air jet was applied to the mucosa, and Nikolsky's sign (Figure 2(a)) was observed. An incisional biopsy was conducted to investigate the blistering area histopathologically. It was a difficult procedure owing to epithelial detachment and fragility (Figure 2(b)). Surgical cement was used as a postoperative dressing (Figure 2(c)). The fragment was sent for histopathological analysis. Drug therapy was initiated as a combination of prednisone 30 mg/day, for ease in possible adjustments, and topical clobetasol propionate 0.5% cream twice a day during one month. After the treatment, 45 days since the patient first came to the office, there was an improvement in pain and the blistering of the gingiva. However, the gingiva remained erythematous (Figure 3). The anatomopathological examination results were compatible with BMMP (Figure 4). Dapsone 100 mg/day was prescribed, which was subsequently reduced to 50 mg/day. The corticosteroid dose was also gradually reduced. The patient's symptoms steadily improved, with no apparent pain and bleeding. (Figure 5).

## 3. Discussion

Several autoimmune diseases can affect the oral cavity. Pemphigus vulgaris and the pemphigoid family are the most common dermatoses associated with oral lesions [6].

According to the literature, stress can damage the immune system, contributing to autoimmune diseases [9]. Our patient reported that the symptoms started after a significant emotional shock and that she was under postmenopausal hormone treatment. Hormone treatment has been reported to interfere in immunosuppressive activity, changing the immunological capacity of women and leading to a number of emotional symptoms [10].

As for BMMP and its clinical signs, some studies have shown that desquamative gingivitis can be present in up to 95% of cases [5, 11, 12]. Erosive flat lichen and, less frequently, pemphigus vulgaris may also cause desquamative gingivitis [5]. Periodontists have a key role in diagnosing, preventing, and treating complications of these conditions [4]. The changes caused by BMMP in the oral epithelium affect periodontal health, facilitating the accumulation of dental biofilm and changing periodontal parameters. Bleeding, probing depth, and the clinical insertion level may make diagnosing the condition challenging due to the overlapping clinical signs of different autoimmune diseases and periodontal changes [3, 11, 12].

The common BMMP shows a histological separation of the epithelial layer with the presence of an inflammatory infiltrate [13]. A direct immunofluorescence test also can be performed to detect immunoglobulins, complementing the biopsy [13–15].

With regard to treatment, corticosteroids are the first choice, while localized lesions can be treated with fluocinonide 0.05% and more severe lesions with clobetasol propionate 0.05%. Patients who demonstrate only intraoral lesions usually respond effectively to topical corticosteroids alone [16]. However, using a topical drug could be difficult especially for elderly patients with impaired hand movements. In these cases, low-level laser therapy would appear to be a possible treatment for some elderly patients with oral erosive lesions due to mucous membrane pemphigoid [17]

Depending on the extent and severity of the lesions, systemic administration of corticosteroid immunomodulators [6] may also be indicated. These act directly on the body's defense system, preventing the production of antibodies that attack the epithelial tissue forming the blisters. In more severe cases, the elimination of gingival irritative factors such as dental biofilm and calculus should also be treated with the association of drug therapy [5]. As it is a dermatosis, multidisciplinary follow-up with a dental surgeon and dermatologist is essential [12].

## 4. Conclusion

To date, the BMMP case reported in the article has been successfully treated with systemic and topical immunosuppressive therapy. The efficacy of systemic corticosteroids with dapsone has been highlighted and can lead to the stabilization and adequate control of the disease.

History and a detailed clinical examination are essential and should be associated with a histopathological examination when necessary to achieve the correct diagnosis and treatment.

## Data Availability

The [PDF-Histopathological report] data used to support the findings of this study are included within the supplementary file.

## Additional Points

*Summary*. The dental surgeon has a vital role in diagnosing benign mucous membrane pemphigoid and should use auxiliary examinations as well as know the association of systemic corticosteroids with dapsone.

## Supplementary Materials

Supplementary Materials Histopathological analysis: histopathological result represented by submucous tissue with subepithelial detachment. The subepithelial inflammatory process is small. The histopathological board may be compatible with subepithelial bullae, type bullous pemphigoid. Clinical correlation is recommended.
