# Supplementary material for: Benign Mucosal Membrane Pemphigoid as a Differential Diagnosis of Necrotizing Periodontal Disease
Source: Case Rep Dent. 2020 Dec 22;2020:8885158. doi: 10.1155/2020/8885158 (PMC7772047; doi:10.1155/2020/8885158)
Supplement: Supplementary Materials — Histopathological analysis: histopathological result represented by submucous tissue with subepithelial detachment. The subepithelial inflammatory process is small. The histopathological board may be compatible with subepithelial bullae, type bullous pemphigoid. Clinical correlation is recommended. [file 8885158.f1.pdf]

CENTRO DE PATOLOGIA DE CURITIBA

Dr. Luiz Fernando Bleggi Torres CRM 7287
Dra. Elizabeth Schneider Gugelmin CRM 7828
Dr. Luiz Martins Collaço CRM 8402
Dra. Lúcia de Noronha CRM 14547
Dra. Ana Paula Martins Sebastião CRM 15282
Dra. Ana Cristina Lira Sobral CRM 19283

Unidade : Serviço de Patologia - Hospital das Nações | *Exame nº* **15/B00471**

Nome : **Elizabete Alves Nedbayluk** | *Idade* : **50a1m**
Origem : **(E)** | *Complem.* :
Requis. : **Dr. Lucimari Teixeira** | *Data* : **15/05/2015**
CRM : **23053** | *Prontuario* :

Conv. : **Sinam** | *Matríc.* : **c-12972**

Procedimento(s):

Boca:
Peça Convencional Simples 4.06.01.20.0 x 1

Relatório Médico:

Resumo Clínico:
Paciente com hipótese diagnóstica pênfigo bolhoso.

Macroscopia: Data: 15/05/2015
Material: ressecção lesão em gengiva.

Um fragmento irregular de tecido rotulado como de gengiva, medindo 0,7x0,2x0,1cm. Coloração creme, aspecto liso e brilhante, consistência macia e elástica. 1/4 TI.

Microscopia: Data: 02/06/2015
Cortes histológicos corados por hematoxilina-eosina demonstram fragmentos teciduais com características arquiteturais e celulares que corroboram o diagnóstico a seguir:

Conclusão: Data: 02/06/2015

QUADRO HISTOPATOLÓGICO REPRESENTADO POR TECIDO SUBMUCOSO COM DESTELHAMENTO DA LESÃO BOLOHOSA QUE É SUBEPITELIAL. O PROCESSO INFLAMATÓRIO SUBEPITELIAL É ESCASSO. O QUADRO HISTOPATOLÓGICO PODE SER COMPATÍVEL COM BOLHOSE SUBEPITELIAL TIPO PENFIGOIDE BOLHOSO. RECOMENDAMOS CORRELAÇÃO CLÍNICA.

Curitiba, 02/06/2015

Lúcia de Noronha

Dra. Lúcia de Noronha
CRM: 14547
